# Viral Diseases in Water Buffalo (*Bubalus bubalis*): New Insights and Perspectives

**DOI:** 10.3390/ani14060845

**Published:** 2024-03-09

**Authors:** Julio Martínez-Burnes, Hugo Barrios-García, Verónica Carvajal-de la Fuente, Belkis Corona-González, Dasiel Obregón Alvarez, Dora Romero-Salas

**Affiliations:** 1 Animal Health Group, Facultad de Medicina Veterinaria y Zootecnia, Universidad Autónoma de Tamaulipas, Victoria City 87000, Tamaulipas, Mexico; vcarvajal@docentes.uat.edu.mx; 2 Instituto de Ecología Aplicada, Universidad Autónoma de Tamaulipas, Victoria City 87000, Tamaulipas, Mexico; 3 Departamento de Salud Animal, Centro Nacional de Sanidad Agropecuaria (CENSA), San José de las Lajas 32700, Cuba; bcorona@censa.edu.cu; 4 School of Environmental Sciences, University of Guelph, Guelph, ON N1H 2W1, Canada; dasieloa@uoguelph.ca; 5 Laboratorio de Parasitología, Facultad de Medicina Veterinaria y Zootecnia, Universidad Veracruzana, Colonia Unidad Veracruzana, Veracruz 91710, Veracruz, Mexico; dromero@uv.mx

**Keywords:** water buffalo, *Bubalus bubalis*, health, viral diseases

## Abstract

**Simple Summary:**

Buffaloes are typically raised in tropical, flood-prone zones, thus exposed to conditions conducive to the growth of disease-causing pathogens and their vectors. They generally share the habitat with cattle, representing sanitary problems because viral agents can transmit between both species; many are zoonotic and public health concerns. The mobilization and introduction of buffaloes and their biological products from one continent or country to another pose a risk of spreading viral diseases considered exotic in certain regions. This underscores the critical need for rigorous epidemiological surveillance of infectious diseases within buffalo herds. Additionally, it highlights the potential implications and risks these diseases pose to other livestock species and public health in newly inhabited regions. This review discusses the more reported viral diseases in water buffaloes, their characteristics, epidemiology, disease behavior, and interaction with other species, vectors, and pathogens. The epidemiology of buffalo viral diseases must be continuously reconsidered and updated to obtain better prevention and control programs.

**Abstract:**

The water buffalo (*Bubalus bubalis*) has great adaptability to rustic environments and more variable conditions than cattle, who generally share the habitat. Diseases carried by buffaloes are relatively unknown and ignored and could be transmissible; an imbalance occurs between pathogens, environment, and susceptible hosts, generating a severe animal health problem. Also relevant is the effect of climate change on the populations of vectors that transmit viral diseases. The discovery of new virus variants that can pass from bovine (Bos) to buffalo or vice versa or to humans has highlighted the relevance of viruses crossing the host barrier. This review discusses the clinical viral diseases most reported in the water buffalo, characteristics, epidemiology, and recent findings about disease behavior, interaction with other species, the host, vectors, and pathogens. Diseases reviewed include Foot and Mouth Disease, Rinderpest, Malignant Catarrhal Fever, Infectious Bovine Rhinotracheitis, Bovine Viral Diarrhea, and Rabies. Also, vector-borne diseases include Lumpy Skin Disease, Ephemeral Fever, and Blue Tongue. The review also considers emerging viruses such as Buffalo Pox and Schmallenberg and, finally, other viruses such as papillomatosis. The knowledge and epidemiology of buffalo viral diseases must be constantly reconsidered and updated for adequate prevention and control programs.

## 1. Introduction

Buffaloes (*Bubalus bubalis*) are native to Asia, and they were taken to Africa, Europe, Oceania, and later to the American continent to the United States, Venezuela, Argentina, and Brazil [1,2,3]. The buffalo population has multiplied and is currently found in all American countries [4]. The FAO reported the world population as ∼206.6 million in 2018. Minervino et al. [4] identified 77 countries with buffalo herds, estimating 208,098,759 head. Most of the buffaloes are in Asia (96.7%), followed by Africa (1.6%), America (1.23%), Europe (0.22%), and Oceania (0.07%), Since the 1970s, the number of buffaloes in the world has increased by 91%, five times more than that of cattle. Latin America has the highest growth in buffalo production systems worldwide. The most numerous herds are found in decreasing order in Brazil, Venezuela, Colombia, Argentina, Cuba, and Mexico [4]. In Mexico, the arrival of the buffalo was between 1996 and 1998, coming from Belize but of Asian origin. According to information from the Mexican Association of Water Buffalo Breeders (AMEXBU), there are currently herds in 21 states [5], with a total of 45,000 head [4]. Therefore, the introduction of buffaloes to Mexico occurred relatively recently, and much is still unknown about their demographic and epidemiological situation.

The water buffalo has great adaptability to rustic environments, provides an excellent conversion of vegetables of low nutritional value into better quality meat and milk, and is well suited for work, which makes it very attractive to producers [3,6]. Its morphological and physical characteristics facilitate greater adaptation to more variable conditions than cattle [2,7]. The production of buffaloes is a viable alternative in areas unsuitable for cattle due to low, flooded, or swampy terrain, and natural grasslands of low nutritional value in tropical and subtropical climates. The advantages of this livestock species stem from its physiological characteristics, such as enhanced longevity, rusticity, extended chewing duration, efficient ruminal processing, disease resistance, and brief anestrus periods.

Buffaloes often cohabit with cattle, posing health risks as infectious agents can transfer between the two species, leading to a range of diseases that adversely impact production [8]. Many of these pathologies are of infectious origin and are transmitted from one animal to another, and others are zoonotic, infecting humans and generating public health problems [9]. Raising buffaloes and cattle together or with other domestic animals and contact with wild animals in different ecological systems expose the species to diverse pathogens [10]. These arising contagious illnesses induced by viruses, bacteria, fungi, protozoa, and endoparasites have a significant economic impact. The importation and exportation of livestock are significant ways of spreading diseases from one continent or country to another. The behavior of wallowing or submerging in muddy water predisposes buffalo to different diseases. Mixing with other buffaloes, ruminants, and other animals further facilitates disease transmission [10].

Despite the buffalo’s numerous benefits in adapting to different environments, it is essential to remember that in some countries, it is a recently introduced animal whose health status is still unknown or undervalued. In addition, the role that it can play as a reservoir of pathogens can have a transmission effect on other ruminants and public health. A relevant aspect of the coexistence of the water buffalo with other species in shared production systems is that when the diseases that the buffaloes carry or could be susceptible to are unknown, an imbalance occurs between the etiological agents, the environment and susceptible hosts (buffalo and other species), which can generate a severe animal health problem and place the health of producers and people in contact with animals or consumption of their by-products at risk [11]. Therefore, the health-disease concept in buffalo production acquires great relevance. Hence, in this work, we review the clinical viral diseases most reported in the water buffalo, as their characteristics, epidemiology, and recent findings allow a greater knowledge about the health of buffaloes, the behavior of diseases, their interactions with other species, and the interactions of the hosts, vectors, and pathogens that may be of benefit to veterinarians, professionals, and technicians. The reviewed diseases include Foot and Mouth Disease, Rinderpest, Malignant Catarrhal Fever, Infectious Bovine Rhinotracheitis, Bovine Viral Diarrhea, and Rabies. The vector-borne diseases include Lumpy Skin Disease, Ephemeral Fever, and Blue Tongue. This review also considers emerging viruses such as Buffalo Pox and Schmallenberg and other viruses such as Papillomatosis.

## 2. Foot and Mouth Disease (FMD)

An Aftovirus, an RNA virus of the Picornaviridae family, generates FMD involving cloven-hoofed animals with seven main serotypes: O, A, C, SAT 1, SAT 2, SAT 3, and Asia 1, with several immunological subtypes of different virulence. Since 1978, the Asia 1 serotype has been reported to be more severe in buffalo than in cattle. Serotype A has also been detected in buffaloes related to outbreaks in Southeast Asia [12]. Serotypes O, A, and Asia 1 are endemic in India, with O responsible for more than 80% of FMD outbreaks in that country [13].

Asia-1 is the most prevalent serotype in Pakistan, followed by A and O. Phylogenetic investigation revealed numerous distinct clusters of serotypes Asia-1 and A. The study indicates a high prevalence of subclinical FMDV infection in vaccinated buffalo in Pakistan and highlights the importance of that undetected subclinical infection in FMD surveillance in endemic regions [14]. Molecular characterization of the circulating FMDV strains associated with a recent outbreak in Egyptian water buffaloes revealed that the circulating virus was SAT-2 serotype, closely related to the lineage of lib12, topotype VII, with a similarity of 98.9%, explaining the high morbidity of FMDV outbreaks in Egypt [15]. Phylogenetic and sequencing analyses demonstrated that serotype O, EA-3 topotype, VP1 is the prevalent serotype responsible for FMDV lesions and high mortality in Egypt’s young calves, adult cattle, and water buffalo [16]. Status of Botswana from 2006 to 2022: SAT2 topotypes I, II, and III and SAT1 topotype I were responsible for most outbreaks in livestock, including buffaloes [17]. In Egypt, the emerging FMDV serotype A, the Europe-South America (Euro-SA) topotype, was recently detected during severe outbreaks that affected several farms, with higher mortalities in water buffalo than in cattle [18].

FMD is almost everywhere, excluding North and Central America (north of Panama), Australia, New Zealand, Japan, Great Britain, and Scandinavia. European Union (EU) countries are usually free [19]. Severe cases have been described in native swamp buffalo from India, Egypt, Romania, and South America [20,21]. For North and Central America, it is considered exotic and is one of the main objectives of prevention and control strategies for its entry.

FMD is an acute and highly transmissible infection that can disseminate to extensive areas due to the movement of infected or contaminated animals, products, objects, and individuals. The susceptibility of buffaloes to FMD varies by country and virus strains. Cattle and buffalo are mainly infected by aerosol transmission, ingestion, or direct contact. The disease is transmitted during the acute phase, from cattle to buffalo and vice versa [22]. Humans can become contaminated via skin or oral mucosa breaks by managing sick animals, virus in laboratories, or by consuming infected milk. Infection in humans is transient and benign, so it is not deemed a severe public health concern, but due to the number of impacted species, the high infectivity rate, and the fact of virus shedding before clinical signs appear, it is one of the most feared notifiable diseases. In addition, because of the similarity of FMD with other vesicular or ulcerative diseases, it is considered a disease for mandatory notification; its differential diagnosis is essential at the first sign of an unusual outbreak. The incubation period is 2–21 days (average 3–8). The infection (morbidity) rate can reach 100 percent; however, low mortality in buffalo can increase by 20–25%. Persistent infection in buffalo during the first 35 days after infection is similar to that in cattle [22]. The role of the buffalo as an FMD transmitter that can keep the disease persistent in the absence of clinical disease has been confirmed since 2009 [12].

**Figure 1:**
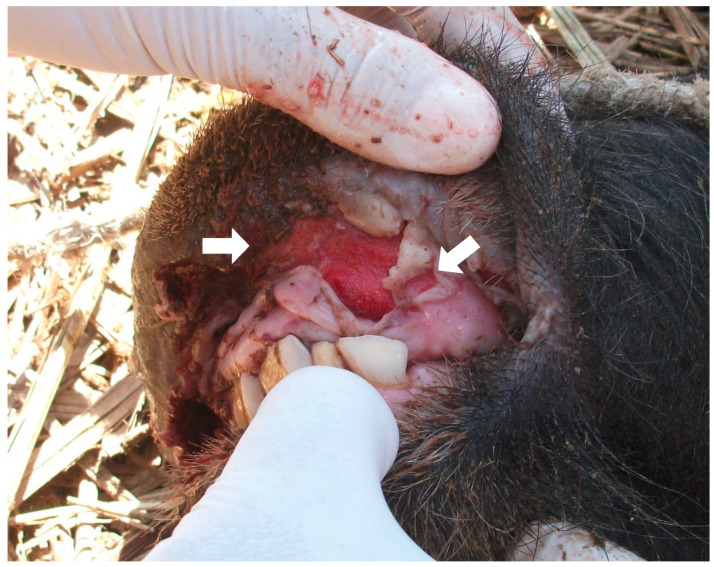
Female buffalo with severe ulcerative lesions on the gums with Foot and Mouth Disease. Images kindly provided by Dr. Karima Akool AlSalihi, Associate Professor, College of Veterinary Medicine/Al Muthanna University, and Dr. Jalil Abed Gatie /Consult/ Veterinary directorate, Ministry of Agriculture of Iran.

Clinical signs of FMD in bovines and buffaloes are salivation, sadness, anorexia, and limpness due to vesicular skin lesions on the lips, tongue, gums, nostrils, coronary bands, interdigital spaces, and nipples (Figure 1). Initially, there is fever and decreased milk production. Vesicular lesions become ulcerative and susceptible to infection. Pathological changes include non-suppurative lymphoplasmacytic myocarditis [16]. FMD has been reported in the Indian buffalo (*Bubalus bubalis*) with characteristics similar to those in cattle, with differences in minor lesions on the tongue and initial scaly lesions on the feet that eventually became vesicular in buffaloes [22]. Lactancy can be diminished by up to 30% in buffaloes. The disease can be more severe in lactating buffalo, with high mortality. However, all age levels are equally affected and sometimes with high mortality [23]. Vesicular and ulcerative eruptions characterize postmortem findings.

Strategies for FMD control depend on the existing health situation. For free countries, eradication implies a norm of non-tolerance to the presence or possible invasion of the virus. In contrast, in countries considered endemic, control implies virus tolerance, but its effects are minimized with vaccination and other animal health actions [24]. Within the control strategies, sanitary slaughter is used, which is applied when outbreaks occur in countries that are free of FMD or as the final stage in an eradication campaign. Control of the movement and importation of animals and products from countries with the disease to other free ones, eliminating the origin of infection (slaughter of all infected populations and those in contact), and epidemiological studies are essential in the eradication. In countries that “live” with the disease, immunization methods have been studied through inoculation twice a year, with a cocktail vaccine containing the specific serotypes circulating locally. The VP1 capsid protein has been proposed by molecular studies as a significant immunogenic site and tested for vaccines with encouraging results [25]. Recently, the first report on the genetic and antigenic variation of FMD virus during virus persistence in cattle and domestic Asian buffalo was published. The proportion of animals from which virus RNA was recovered was not significantly different between cattle and buffalo. However, the virus was isolated from a higher proportion of buffaloes and for a longer time compared to cattle. Authors suggest that this may reflect differences in virus–host interactions between cattle and buffalo that may allow longer survival in buffalo. Alternatively, differences in secretory antibodies (avidity or amount) between cattle and buffalo could decrease successful virus recovery from cattle samples [13]. In India, successful long-term vaccination programs have reduced FMD presentation and losses [26]. However, in Southeast Asian countries, despite many years of vaccination, numerous factors have impeded successful FMD control, including unregulated “informal” cross-border movement of livestock and livestock products, difficulties in implementing vaccination programs, the emergence of new viruses, topotypes, and lineages, low-level technical capacity and biosecurity at the national level, limited farmers’ knowledge of FMD, failure to notify and respond to outbreaks on time, and limitations in national and international FMD control programs [12]. Continuous monitoring and serotyping of the existing circulating FMDV isolate and regular vaccination with reevaluation of the currently used vaccine are recommended in Egypt to prevent the recurrence of outbreaks [16].

## 3. Rinderpest

The causative agent of rinderpest is an RNA virus, family Paramyxoviridae, genus Morbillivirus, characterized by a high rate of morbidity and mortality with virulent strains that in India can reach up to 77%, but variable with mild strains. It affects cattle, water buffalo, sheep, goats, and wild animals. It does not affect humans and is not a public health risk. Buffalo susceptibility is variable: Egyptian and Turkish buffalo appear reasonably resistant, while Far Eastern species appear highly susceptible. Indian buffaloes are three times more susceptible than cattle, possibly due to host specificity to virus strains [27]. The disease has been eliminated from most of the planet. Nevertheless, it has continued to occur in India, Pakistan, the Philippines, and Turkey, countries where buffaloes are raised. The virus has never been reported in the Americas, Australia, or New Zealand [20]. The virus is transmitted by direct and indirect contact through secretions, urine, feces, vaginal discharge, and milk. The main place of attack is the epithelium of the upper or lower respiratory tract. The incubation period in buffaloes is 3–7 days, but this can vary due to differences in innate resistance [28].

Clinical findings of rinderpest in buffaloes include fever (40–42 °C), depression, anorexia, reduced rumination, rough coat, and increased respiratory and heart rates. After two to three days, there is congestion of the mucous membranes, intense mucopurulent tearing and abundant salivation, anorexia, necrosis, and erosion of the oral mucosa. Subsequently, profuse hemorrhagic diarrhea with mucus and necrotic debris occurs. In addition to severe tenesmus, dehydration, abdominal pain, abdominal breathing, weakness, decubitus, hypothermia, and death can occur within seven to twelve days [20]. A peracute form has been described in young and neonatal animals, and an atypical form even with abortion and nervous signs. Because of the virus’s lymphotropic effect, latent infections exacerbate susceptibility to other infectious agents. The characteristic lesions are hemorrhages, necrosis, erosions in the mouth, intestine, respiratory tract, lymphadenomegaly with edema, dehydration, and emaciation [20].

Since there is no specific treatment, only supportive treatment for diarrhea and fluid loss, sanitary prophylaxis should be used, with isolation or sacrifice of ill and in-contact animals, destruction and proper disposal of carcasses and infective material, and protection of unaffected areas. For free countries, the same strategies to avoid introduction where Foot-and-Mouth Disease is mentioned as an exotic disease would be applicable [20]. In areas considered epizootic, the attenuated virus strain vaccine is used, which delays immunity but lasts for life, or it can be readministered in problem areas. The OIE also recommends heat-stable recombinant vaccines [29]. The Global Rinderpest Eradication Program (GREP) was initiated in the 1980s [28]. GREP was coordinated by the Food and Agriculture Organization of the United Nations (FAO) in collaboration with the WOAH and major donors such as the European Commission, and progressively reduced the number of countries with the disease; the last outbreak was in Kenya in 2001. The world was officially declared free of rinderpest in 2011, and it is the first animal disease to be completely eradicated in human history [30]. However, it is imperative to consider it in the differential diagnosis of ulcerative and erosive diseases since it is mandatory to report them and to continue considering all measures and strategies to avoid their reappearance and entry into different countries.

## 4. Malignant Catarrhal Fever

Malignant Catarrhal Fever (MCF) is caused by a lymphotropic herpes virus of the Orthoherpesviridae Family, subfamily Gammaherpesvirinae, Genus Macavirus with two species or strains: *Macavirus alcelaphinegamma1* (AlGHV1) and alcelaphine gammaherpesvirus 2 (AlGHV2), endemic in wildebeest and antelope, respectively. In addition, a sheep-associated form (SA-FCM) identified as *Macavirus ovinegamma2* (OvGHV2) is endemic in sheep and represents a worldwide problem in cattle and buffalo, as well as the form recently associated with *Macavirus caprinegamma2* (Cp GHV-2), endemic in domestic goats [31]. It is a pansystemic, frequently fatal disease that occurs as a complex of syndromes that affect ruminant cattle, water buffalo, American bison, and cervids.

The principal carriers and their viruses are sheep (ovine herpesvirus-2), wildebeest (alcelaphine herpesvirus-1), and goats (caprine herpesvirus-2). Another strain of unidentified origin causes MCF in white-tailed deer. Virtually all clinical cases are caused by the sheep or wildebeest viruses.

Regarding epidemiology, AlHV-1-associated disease can be seen in zoos worldwide and areas of sub-Saharan Africa that contain wildebeest and is the most important MCF virus in parts of Africa. However, OvHV-2-associated disease can also be seen. In contrast, OvHV-2 is the main cause of MCF in animals outside of Africa [31]. In the African form in wild animals, the virus can be transmitted from a latent infection by the African antelope, which includes the wildebeest, grassland antelope, and the topi or Damalisco, and occurs in the wild, zoos, and parks. Domestic and wild sheep and goats are also considered virus reservoirs [32]. Sheep-associated MCF occurs worldwide, and immunosuppression factors are necessary as precursors of this form [33]. A cross-sectional study on the prevalence and molecular detection of OvHV-2 in apparently healthy buffalo in Pakistan confirmed that they could be carrying OvHV-2 acquired from OvHV-2-positive sheep and goats [34].

There is no evidence of transmission to humans. Sheep-associated MCF is of great economic concern in Indonesia, where water buffaloes are commonly housed with sheep and goats [35].

MCF affects all ages and breeds of buffaloes, which are more susceptible than cattle, with a morbidity of 20–50%, and it is common in late winter/spring [23]. The prognosis of MCF is unfavorable; once clinical signs are observed, mortality in buffalo is usually 75% to 100% [31]. The incubation period can be up to 200 days. The disease has been reported in most buffalo-rearing countries [20]. There are reports of MCF in swamp buffalo in New Zealand [36,37], in Indonesia [38], and in Thailand in association with sheep [39]. In the Americas, there is a report of a single outbreak in Murrah buffaloes in Brazil with the OHV-2 virus [33], kept with cattle and sheep, with high mortality and a trend of a single outbreak, similarly to what occurs in other countries [32].

MCF is an unpredictable disease; cases sometimes occur in animals exposed to carriers but without incident for years. It is unclear why animals do not always become ill upon exposure to carriers. However, stressors that increase virus shedding in the carrier and susceptibility in the incidental host, environmental conditions (high humidity) that increase survival or concentrate virus, or unusually high virus replication, have been suggested as possible factors. Pregnant and younger animals appear to be more susceptible.

Classic MCF presents in cattle with fever, ocular and nasal discharge, panophthalmitis, corneal opacity; generalized lymphadenopathy; arteritis, inflammation, and necrosis of the digestive, respiratory and urinary mucosa; dermatitis; neurological signs and arthritis [20,32]. In cattle, four clinical forms are described: Peracute, intestinal, head and eyes, and mild. In buffaloes, the disease is seen in the head and eyes or in intestinal forms [23]. In Indonesian buffaloes, skin hyperemia, lymphadenomegaly, and depression have been described [38]. Typical signs in buffaloes include anorexia, high fever, depression, and poor body condition, with necrosis and erosive-ulcerative oral cavity lesions. Neurological signs include ataxia and severe seizures leading to death. Diarrhea and hematuria are also seen in buffalo [36,37]. Other reports in Thailand describe the same lesions and severity in buffaloes and cattle, with generalized vasculitis with fibrinoid degeneration [39]. Signs and lesions in water buffaloes are similar to those in cattle, except that fibrinoid arteritis is uncommon, oral and esophageal lesions are rare, keratitis is relatively mild with inconsistent corneal opacity [31,39], and epicarditis-myocarditis is usually present, with myocardial necrosis [38,39]. In the Americas, it has been described in an outbreak of MCF in buffaloes in Brazil that clinical manifestations and lesions were similar to those previously described in cattle and from various geographical locations with MCF due to OvHV-2 and in Africa associated with AlHV-1 [40]. The authors confirmed the association of OvHV-2 in this outbreak [33]. This is one of the few studies from Brazil that have identified OvHV-2 DNA in sheep during MCF outbreaks [40]. Based on signs and lesions of FCM, it is necessary to consider it in the differential diagnosis of vesicular and ulcerative diseases, in addition to being mandatory to report due to risk.

Regarding prophylaxis, there is no effective vaccine for MCF. Experimental killed virus vaccines are not consistent in generating protection under challenge; however, some have induced significant virus-neutralizing antibody titers. Also, there is a prospect for a future modified live virus vaccine. In addition, there is no specific treatment and only support [20]. Therefore, disease control is possible by separating cattle and buffalo from possible reservoirs, such as sheep, goats, and wildebeest, especially during calving seasons. Repopulation of cattle ranches with antelope alcelaphines, wild sheep, or goats should be discouraged. In either case, a negative MCF serological test or a negative PCR test should be required for any wild ruminants before they are placed in or transferred between zoos to prevent the introduction of potential virus carriers. The immediate separation of the bovine or susceptible sheep and goats host or the alcelaphine or wild ruminant is mandatory to contain an outbreak in buffaloes [20].

## 5. Infectious Bovine Rhinotracheitis

Infectious Bovine Rhinotracheitis or Infectious Pustular Vulvovaginitis (IBR/IPV) is an infectious disease of cattle due to the bovine herpes virus 1 (BHV-1 or BoV-1H), belonging to the family Herpesviridae, subfamily Alphaherpesvirinae. Buffalo Alphaherpesvirus was differentiated from BHV-1 on restriction profiling of viral DNA in Australia, indicating that it might be a different species [35]. Studies in Italy established that buffaloes are exposed to both BHV-1 as well as Bubalino Herpes Virus 1 (BuHV-1) at the population and herd level but not associated with mixed herds, with a high prevalence of coexistence of the two viruses at the herd level [41,42]. Mortality is low, but economic losses can be considerable.

BHV-1 can infect the upper respiratory or reproductive tracts and disseminate via monocytes, other leukocytes, and peripheral nerves, evoking latent infection in neuronal cells of the trigeminal and sacral ganglia. Latent infection permits the virus to prevail in infected hosts for undetermined periods. Reactivation can occur spontaneously or be induced by natural or artificial immunosuppressive stimuli (labor, transport), leading to virus replication, re-excretion, and environmental spreading [20].

IBR-susceptible species are domestic and wild cattle. Transmission occurs through aerosols, direct and indirect, contact and venereal, artificial insemination, respiratory, eye, and reproductive secretions (amniotic fluid, placenta, fetus, and semen) of infected animals, for ten to fourteen days after infection, even asymptomatic, and also through milking machine suckers [35]. The incubation period is from two to twenty days.

The distribution of the disease is worldwide. The virus was first isolated in Australia (1972), then in Malaysia, India, and Egypt. In India, seropositivities from 7.1% to 58% to BHV-1 have been described in buffaloes [20,43,44]. In Italy, serological investigations have shown seroprevalences ranging from 59 to 82% in herds and 30 to 80% in animals, with a more active viral circulation when buffalo and domestic cattle are raised together [20]. The first experimental transmission of BoHV-1 in buffaloes was carried out in Italy in 2010, confirming its susceptibility to infection and the buffalo’s possible role as host/reservoir of the virus [45]. Also, in Italy, the first virus isolation of BoHV-1 was described in cases of natural infections in buffaloes with rhinotracheitis and abortions in 2011 and 2012 [46].

In 2012, investigations of buffalo herds from a farm in central Italy (Marche region) with severe clinical respiratory symptoms in young buffaloes showed that one young buffalo’s viral isolation from nasal swabs was characterized by PCR and genomic sequencing, resulting in a 100% homology with the glycoprotein E (gE) of Bubaline herpesvirus type 1 (BuHV-1). The study concluded that BuHV-1 was detected in young buffaloes with severe respiratory clinical symptoms for the first time [47].

In the same country, seroprevalences of 59% have been described in buffaloes with higher seropositivity to BuBHV-1, which is why it is considered the main circulating infection of alphaherpesvirus with an impact on mobilization and commercialization [48]. In a recent study in southern Italy, using real-time PCR, fetuses of 14 water buffaloes that showed abortions were positive for BuHV-1 (4 animals) and/or BoHV-4 (11 animals), with one of these cases showing co-infection, reporting the first identification of BoHV-4 in water buffaloes [49]. Studies in Switzerland provided evidence for the potential roles of water buffaloes in the epidemiology of ruminant pestiviruses and possibly bovine alphaherpesvirus 2, indicating that water buffaloes are susceptible to interspecies viral transmission and may act as intermediate hosts or even as reservoirs for these viruses [50].

Studies in Brazil describe antibodies against IBR/IPV in 59% of buffalo sera using the ELISA test [51] and by micro serum neutralization of 14.7% [52]. The first report of the isolation and molecular characterization of BuHV1 in buffaloes in South America, according to Maidana et al., was in 2014 in Argentina [53]; it was suggested that two different lineages of BuHV1 circulate in the buffaloes of that country. In Mexico, the first report of serological evidence of BHV1 in buffaloes was in Veracruz, with 57.6% of animals seropositive over seven years of age in five ranches. Cohabitation with cattle was an important factor for the transmission of bovine herpesvirus in water buffaloes [54]. A second study in 368 buffaloes showed a seroprevalence of 59%, with a higher prevalence in those older than seven years (86%) and in females with a history of abortion, although without apparent risk of interaction with cattle [55].

**Figure 2:**
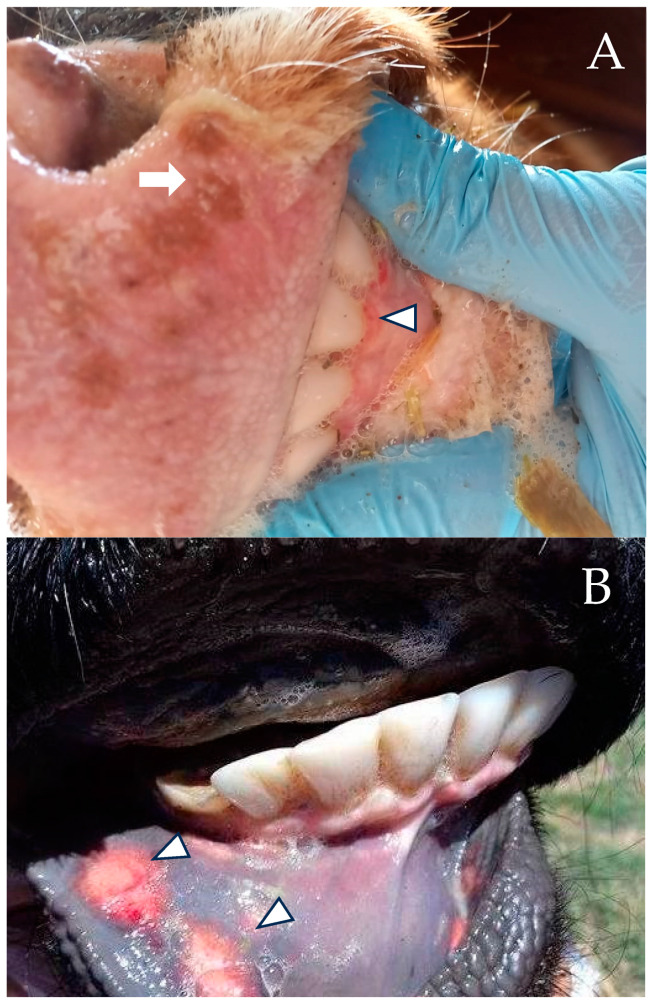
(**A**) One-to-two-year old female water buffalo with ptyalism and multifocal erosive lesions on the nose (arrow) and gums (arrowhead) caused by IBR at a ranch in Veracruz, Mexico. The female and the dam tested serologically positive for IBR. (**B**) Female with multifocal ulcerative stomatitis (arrowheads) at the same ranch in Veracruz, Mexico.

The respiratory form is characterized by fever (up to 42 °C), diminished milk production, anorexia and emaciation, severe hyperemia of the nasal mucosa (red nose), with multifocal necrosis (Figure 2A,B), serous discharge from nose and eyes, conjunctivitis, hypersalivation, mastitis, cough and, infrequently, death from obstructive bronchiolitis or secondary bacterial bronchopneumonia. The respiratory form can be complicated by abortive one with late abortion (between the fifth and eighth month of gestation) and retained placenta, which may be the only manifestation. In the genital form, the disease lasts two to three weeks with moderate fever, hyperemia of the genital mucosa with 1 to 2 mm vesicles, white discharge from the vulva, pollakiuria, and decreased milk production, and males suffer balanoposthitis. The disease is more severe in calves under six months, with diarrhea, meningoencephalitis, blindness, and high mortality [56]. Due to the type of BHV-1, lesions in the mucosa should be considered in the differential diagnosis of other viral ulcerative diseases included in this review, mandatory reporting, and exotic for some countries.

Regarding prophylaxis, there is no specific treatment; broad-spectrum antibiotics can prevent secondary bacterial bronchopneumonia. Once the virus is in a region, it is difficult and expensive to eradicate since animals become inapparent carriers when the disease is established. Routine testing and elimination of positives have been successful in some countries.

Because BoHV-1 and BuHV-1 are genetically related, it is difficult to differentiate these two infections during IBR control/eradication programs. There are no indirect tests accurate enough to differentiate BuAHV-1 and BoAHV-1 infection; seroneutralization does not differentiate them. Recent studies have explored the potential of a newly developed glycoprotein E (gE)-enzyme-linked immunosorbent assay (ELISA) test to differentiate both based on their specific infection status [48].

Different types of inactivated vaccines for BoHV-1 are available, but free countries restrict their use. However, only Austria, Denmark, Finland, Switzerland, and Sweden have eradicated it [20]; in others, eradication is not considered necessary or a priority. However, no commercial vaccine has been developed to date for the control of BuHV-1. Marker vaccines lacking one or more genes in charge of glycoprotein or enzyme synthesis are also used and enable differentiation of vaccinated animals, called Differentiating Infected from Vaccinated Animals (DIVA). Deleting the gene encoding glycoprotein E (gE) of BoHV-1 is the most generally used genetic marker for the BoHV-1 DIVA vaccine. This vaccine facilitates the differentiation of immunized animals (gE-negative) from infected ones with wild-type BoHV-1 or vaccinated with traditional non-deleted vaccines (gE-positive) by gE-specific ELISA test. Using the single BoHV-1 gE-deleted marker vaccine to control BuHV-1 in water buffaloes has been shown to result in cross-protection against BuHV-1 [57].

Recent studies in Italy evaluated an IBR marker immunization protocol to vaccine against BoHV-1 in buffalo species to protect against BuHV-1. The study findings indicate that the IBR immunization protocol used to vaccinate cattle against BoHV-1 does not protect water buffalo against wild type BUHV-1 challenge. Also, further studies are necessary to test increasing the dose used in cattle to vaccinate water buffaloes [58].

## 6. Bovine Viral Diarrhea

This disease is caused by the Bovine Viral Diarrhea virus (BVDV) of the Pestivirus genus, family Flaviviridae, and can develop many different variants.

Currently, the genus is split into three species: Pestivirus A (Bovine viral diarrhea virus 1, BVDV-1), Pestivirus B (Bovine viral diarrhea 2, BVDV-2), and Pestivirus H (HoBi-like pestivirus, atypical ruminant pestivirus) [59]. The origin of HoBi-like viruses is unknown. One hypothesis is that they originated in South America and were introduced to other continents through contaminated biological products, such as fetal bovine serum and vaccines. Another explanation is that the emergence of HoBi-like viruses in cattle resulted from a host species jumping from water buffalo to cattle. Furthermore, the virus presents two biotypes: one that produces cytopathic effects (CPE) in cell culture and another that does not (non-CPE). Persistent infections are caused by the non-CPE viruses that likewise induce fetal malformations, while CPE viruses induce mucosal disease (MD) [20,60,61]. The non-cytopathic form is the most common in cattle [62].

The disease caused by these new viruses resembles the clinical presentations associated with BVDV infection [59]. This type of virus has been reported in cattle in Europe, Asia, and America (Brazil) [10]. In addition to cattle, other ruminants are infected with BVD, including water buffalo. The high prevalence of BVDV in many countries is thought to be a result of the ability of non-CPE BVDV to establish lifelong infections after uterine infection earlier in gestation, thus inducing a pool of persistently infected animals, which become immunotolerant and shed the virus [63]. This biotype is the most often isolated, capable of vertical transmission, and responsible for permanent circulation. Its propagation, manifestation, and distribution depend on its properties, transmission modes, animal immune status, and demographic risk factors [20].

Studies on buffaloes in some countries show very low seroprevalences to BVDV, 4.9% in Southeast Asia, or absent [64], and 3.4% in Cambodia [65], assuming that buffaloes are less susceptible than cattle. However, they are small herds, and the risk of being seropositive is associated with increased herd size or no permanently infected calves. In contrast, the prevalence of BVDV in buffaloes in nine Indian states was 23.2% [66]. Studies in Australia from 1993 to 2001 also describe low seroprevalences (4.5%) in buffaloes, showing they are susceptible, but did not identify persistently infected animals [67]. In 2009, subgenotype 1b was identified in aborted buffalo fetuses in Italy [68]. Reports from Brazil describe serological evidence of the presence of BVDV in water buffalo herds from beef and dairy farms, with two different epidemiological situations. The first noted seroprevalences from 39.6% to 51.3%, with very variable titers that follow active infection, in large, open herds, with many management practices. The second, with low seroprevalences from 8.1% to 11.1% and with slight variation in titers, suggests an epidemiological condition of infection stability, but in small, closed herds, with few management practices and no introduction of new replacements [69]. Recently, active infection with BVDV subgenotype 1b was identified for the first time in Brazil’s two herds of dairy buffaloes [70]. Of interest, the virus has been detected in fetal bovine serum from Brazil, Australia, Canada, the United States, and Mexico [59,71]. Studies in Argentina showed high positivity against BVDV-1 and BVDV-2 in buffalo herds. Molecular analyses confirmed the existence of persistently infected buffaloes in serologically negative animals, and the presence of natural coinfection with two different subtypes was confirmed: BVDV (1a and 1b subtypes) and also with both BVDV species (BVDV-1 and BVDV-2) [72]. In Mexico, Medina-Gudiño et al. [73] reported for the first time the molecular detection of BVDV subgenotype 1b, biotype CP in captive water buffaloes. In recent studies in Switzerland [50], seropositive water buffaloes were detected by ELISA and confirmed by VNT. Considering the ban on anti-pestivirus vaccines in that country, results strongly suggest that seropositive water buffaloes on the three farms had been transiently infected with BVDV. The highest antibody titers were measured against BVDV1h, which was the most frequent subgroup in Switzerland prior to eradication, arguing for a bovine source of transmission.

BVDV is described as a persistent disease of the mucous membranes and as a hemorrhagic syndrome. Clinical findings are influenced by host factors such as BVDV immune status (immunocompetence or immunotolerance), gestation period, and the BVDV genotype involved. Clinical signs in buffaloes are milder than those in cattle but similar in chronology. Buffalo calves experimentally infected with BVDV showed a temperature increase of 40 °C, nasal and lacrimal discharge, and diarrhea. Also, erosive and ulcerative stomatitis with congestion of nasal mucosa and gums, and mild leukopenia (neutropenia and lymphopenia) that later became leukocytosis (neutrophilia) were observed from day 15 to 32 [20]. The disease behavior is an acute and persistent infection and, depending on the epidemiological events, can manifest as outbreaks involving large numbers of animals or as a continuous low incidence of cases in herds. The economic impact is due to productive and reproductive losses: low milk production, reduced conception rate, abortions, fetal mummifications, congenital malformations, weak calves, and increased mortality [56].

The findings of new viruses highlight the importance of intensifying prophylaxis measures to control BVDV infection in cattle and also in buffaloes because these species coexist in different countries. The main prophylaxis is prevention since there is no treatment. The most important thing is careful selection, proper diagnosis, and avoiding herd contact with carrier animals. Prevention focuses on good management and a vaccination program, although vaccines achieve only partial protection; in any case, they will decrease the incidence. A modified live virus or killed virus vaccination program should begin between four and six months of age, at the end of colostral protection, and be repeated before breeding. In the absence of characteristic clinical signs associated with BVDV, diagnosis is based on detecting the virus in the laboratory, of antigens or antibodies in serology, or in milk from unvaccinated dairy herds [56].

## 7. Blue Tongue

Bluetongue (BT) is a disease caused by a virus of the Reoviridae family, Orbivirus genus, and biologically transmitted by five species of Culicoides mosquitoes; others consider that at least 20 species may be vectors. Without these vectors, direct or indirect contact between animals does not transmit the virus [74]. The hosts are all domestic and wild ruminants: sheep, goats, cattle, buffalo, dromedaries, and deer, among others. The incubation period varies from 4 to 20 days [20,75]. As of 2020, 28 serotypes had been reported globally, with putative serotypes recently described [75,76].

The distribution of BT is also associated with the distribution of Culicoides vectors. The BT virus can be introduced into new regions by importing infected animals; however, it will only survive if vectors are present [20,74]. BT is a clinical disease in Africa, the Middle East, India, China, the United States, Mexico, and southern Europe. Positive serologic tests have also been found in Southeast Asia, northern South America, northern Australia, the Solomon Islands, and New Guinea, and, in buffalo, in Egypt, Botswana, New Guinea, and India, with seroprevalences as high as 92% [20,76]. A systematic review and meta-analysis of seroprevalence of BT in India in 2653 buffalo samples showed an overall seroprevalence of 35%, with variations across regions. Though buffaloes are susceptible to BTV infection, generally they do not show overt clinical signs of the disease. Since buffaloes are sub-clinically infected with BTV, they may play an important role in BTV transmission and have significant implications in BT control by vaccination in India [77]. In Switzerland [50], seropositive water buffaloes were detected by competitive ELISA, considering that water buffaloes are susceptible to BTV infection and that a natural infection with BTV seems most likely to occur.

In Mexico, there is a history of seropositivity in cattle, sheep, and white-tailed deer, in the latter with seroprevalences of up to 81% but without clinical outbreaks, indicating enzootic stability [78], as occurs in Texas with 90% seropositivity. The first report of clinical hemorrhagic disease in deer in Mexico corroborated by PCR was described by Martinez-Burnes et al. in 2017 [79]. However, there are no reports of buffaloes.

All ruminants are susceptible to BT infection, but clinical disease occurs more frequently in sheep, white-tailed deer, and cattle. Under natural conditions, it may also be present in elk, pronghorn, antelope, deer, mouflon, captive yak, camelids, and elephants [75]. BT induces mucosal inflammation, congestion, swelling, and bleeding. The infection is generally subclinical in cattle and buffaloes. Sheep are the most affected. Disease manifestations can vary and exhibit fever (42 °C), emaciation, inflammation, ulcers, and necrosis in gums, cheeks, and tongue. In a small proportion of cases, cyanotic tongue, redness, and hemorrhages of the coronary band (above the hoof) cause lameness—abortion, congenital malformations and sometimes pneumonia [80]. The responses of buffaloes to disease could rely on the serotype, infecting dose, and the species implicated in infecting the buffalo [56].

Preventing BT in free zones requires quarantine, serological studies, and vector control. In infected areas, health prophylaxis is through vector control. Vaccination prophylaxis with modified live virus or buffered polyvalent vaccines is possible. Autochthonous serotypes obtained from field infections must be preferentially used in the vaccines [75].

Pregnant ewes should not be vaccinated because of the fetal abnormalities and abortion risk [56]. It is a mandatory reporting disease, and its presence or that of antibodies restricts the mobilization or export to free countries.

## 8. Rabies

Rabies is an infectious disease caused by an enveloped RNA virus of the Rhabdoviridae family and Lyssavirus genus that affects the central nervous system (CNS) in various mammalian species [20,81].

Rabies is commonly reported in cattle but infrequently in water buffalo or is underestimated. The main transmitters in these species are blood-sucking bats, particularly *Desmodus rotundus*, which inoculate the virus in saliva [82]. However, some countries, such as India, report cases in buffalo where it is assumed that the rabies virus is transmitted by stray dogs or rabid mongooses [83]. Reports in buffaloes are rare since they defend themselves well against rabid animals. In some countries, no cases have been reported in buffaloes due to transmission by bats, such as in cattle. However, the mortality of the buffaloes is 100 percent. In Punjab, India, an enhanced surveillance for rabies at the state-wide level estimated that 98 (34–294) buffaloes would be expected to be confirmed with rabies annually [84]. Rabies exists in most countries, except on islands with strict quarantine regulations. The incubation period is almost three weeks [20,85].

In recent years, the molecular epidemiology and diagnosis of rabies has been based on the G gene [85]. G glycoprotein plays an important role in viral pathogenicity and neurotropism [86]. Based on this, a phylogenetic analysis of the Chinese buffalo virus was performed compared with other animal isolates. Immunofluorescence confirmed the virus’s presence in a sick buffalo’s brain; it was isolated and named strain Hubei070308, and with G gene sequencing, it was found that the rabies virus has territoriality but is not species-specific [87]. Many amino acid substitutions in the G protein were found among viruses isolated from different animal species or the same species distributed in different geographic regions. These substituted amino acids may explain why the rabies virus can cross the host barrier to infect from one animal species to another [87]. There are reports of rabies in buffaloes in Brazil in a herd in an area considered endemic for bovine rabies; however, the cases were in young animals that had only received a first vaccination against the virus [82]. According to official figures in Mexico, positive rabies cases in buffaloes were reported in 2012 in Veracruz and 2013 in Oaxaca.

The clinical signs of rabies in buffalo resemble those in cattle. A paralytic form (drooling, gnashing of teeth, tail wagging, anorexia, rigidity of the hind legs, paralysis, decubitus, death in two or three days) or a furious form (hypersensitivity, sexual arousal, dysphagia, rubbing or hitting the forehead against the ground and other inanimate objects such as troughs, loud bellowing, collapse, and death) can occur [56]. In clinical nerve paralysis or ataxia, rabies should be considered in the initial diagnosis, and the official diagnosis by immunofluorescence, histopathology, mouse inoculation, or molecular techniques should be confirmed according to the health guidelines of each country [82].

In those countries where rabies is considered to be transmitted by canine or wildlife bites, wildlife around animal farms should be controlled, and dogs and cats vaccinated [23]. In countries with blood-sucking bats, such as South America, Central America, and Mexico, vaccination campaigns for cattle and buffalo herds should be followed, as well as official control of populations of bats with the presence of the virus, not only to avoid losses in livestock but also because it is an important zoonosis.

## 9. Bovine Ephemeral Fever

A virus of the Ephemerovirus genus of the Rhabdoviridae family causes Bovine Ephemeral Fever (BEF). It falls under the arbovirus category, known as “Three-day disease or Three-day fever”. It is an arthropod-borne viral disease of cattle and buffalo with subclinical involvement in various ruminant species. By phylogenetic analysis based on the Glycoprotein gene, BEFV strains are divided into four clusters: Middle East, East Asia, South Africa, and Australia [88]. It has been suggested that cervid species may serve as important reservoirs for transmitting the disease [89], which manifests clinically by the sudden onset of fever, lameness, rigidity, and spontaneous recovery in three days [23,90,91].

BEF virus is transmitted by biting insects, particularly mosquitoes, mainly *Culex annulirostris*; however, the virus has been isolated from eight species of Cullicoides—sucking mosquitoes. Climatic conditions influence the distribution of insects and the pattern of disease spread and time of onset. Therefore, most cases occur in hot and humid conditions. Rainfall, prevailing wind patterns, streams, or other ground water sources have a significant effect on its occurrence, particularly in wet and dry seasons [88].

The subtropical and temperate regions of Africa, Asia, Australia, and the Middle East have had the main BEF epidemics [90,91]. The disease is unknown in New Zealand, Europe, and the Americas [92]. Mortality is low (1 to 2%), but there are reports of mortality or slaughter of up to 10 to 20%. However, morbidity is considerable (100%), leading to enormous economic losses in production and disruption of national and international trade that generates a variety of complications that have drawn attention to this disease [20,92].

Clinical findings in BEF include sudden onset with fever (41 °C) and severe drop in milk production. Abortion can occur in advanced stages of pregnancy. Animals stop eating and drinking and become depressed, with drooling and a runny nose. The swallowing reflex may be affected, resulting in aspiration of food or water and the development of pneumonia. Also, lameness, typical laminitis posture, muscle swelling in shoulders, back, and neck, and arthritis with serofibrinous exudate are characteristic of the disease [92]. Tremors, rigidity, and clonic muscle movements also occur [23]. By the third day, the affected animal is usually on its feet again and will begin to eat. However, the limp and weakness may last another two to three days. Adults are more affected than calves under six months. Milk production should return to near normal after three weeks, but affected cows late in lactation often dry out. Sometimes mastitis develops.

Analyzing the prophylaxis of BEF, high-producing bulls and cows should receive supportive treatment to reduce temperature and broad-spectrum antibiotics to prevent secondary bacterial complications. Live and inactivated vaccines against BEF are available. The live vaccine gives at least 12 months of protection in cattle, following two doses; the killed one only gives about six months’ protection. Then, buffalo calves can be vaccinated and revaccinated at six months of age to maintain their immunity [56]. Despite its significant impact, BEF remains underreported and underestimated. For a more comprehensive global understanding of BEF outbreaks, it is recommended to list it as a notifiable disease with the World Organization for Animal Health. This not only eases the reporting process but also facilitates the evaluation of vaccine effectiveness and the advancement of diagnostic methods [90]. While the epizootiology of BEF has been thoroughly explored in certain areas, considerable gaps in knowledge exist regarding its distribution, prevalence, and consequences across large regions of Africa and Asia. Few virus strains from these areas have been retrieved and sequenced.

Additionally, the specific vectors responsible for transmission remain undefined globally. Such gaps hinder our understanding of the disease’s potential spread, mainly due to factors like wind-driven vector dispersal influenced by climate change or livestock movement. It also challenges our ability to evaluate the risk of BEF becoming an enzootic disease in regions like Europe or America through local transmission vectors [91].

## 10. Buffalopox

Buffalopox virus (BuffaloPox) (BPXV) is a member of the genus Orthopoxvirus, subfamily Chordopoxvirinae, family Poxviridae. BPXV is a close variant of the vaccinia or vaccine virus. Recent genome data show that BPXV shares a more recent common ancestor of the vaccinia virus (VACV) Lister strain, which had been used to inoculate buffalo calves to produce a smallpox vaccine. Over time, VACV evolved to BPXV upon establishment in buffalo to become increasingly pathogenic for this host and cause infections in cattle and humans [93].

The buffalopox virus was first isolated in India in 1967, and the virus continued to cause sporadic outbreaks in Asian buffaloes in Bangladesh, India, Indonesia, Pakistan, Egypt, Russia, and Italy [94]. The same year, the disease was recognized by the Joint FAO/WHO Expert Committee on Zoonoses as an important zoonotic disease (FAO, 1967). Forty years later, the buffalopox virus became an emerging contagious viral zoonotic disease infecting milkers with high morbidity (80%) [93]. Coupled with the current SARS-CoV2/COVID-19 pandemic, BPXV infections in India depict the vulnerability of humans to the emergence. Growing OPXV infection incidences are being registered worldwide: BPXV in Asia, VACV and VACV-like virus (VLV) in Brazil [95], MPXV in East and Central Africa and the US, and AKPV and ECTV-like OPXV, which are being described at an increasing rate [93]. The first description of isolation and clinical disease caused by vaccinia virus in buffaloes in South America was reported in 2019, corroborating previous serological and molecular studies, and referred to as vaccinia virus Pernambuco (VACV-PE) [96].

Therefore, the epidemiology of the buffalopox virus should be reconsidered more than 30 years after the cessation of the eradication campaigns for human smallpox.

Buffalopox’s clinical signs are similar to those of VACV infections. They are characterized in the soft form by focal skin inflammation (pustular with central necrosis) on the muzzle, udder, nipples, inner thighs, scrotum, ears, and eyes. It can also evolve into a severe systemic and cyclical form with diffuse lesions in particular cases [93]. Morbidity can be very high (70%), but mortality is low [56].

Although not a frequent disease, it is economically relevant in countries where buffaloes are raised. It harms the dairy industry due to the 40 to 70% reduction in the productivity of affected milking animals when it affects the udder and teats, which can lead to mastitis [56,93]. Individuals in close contact with infected animals can contract the virus, resulting in smallpox lesions primarily on the forearms, back of the hands, fingers, wrists, thumbs, right preauricular area, right jaw angle, right side of the nose, and forehead. These symptoms might be accompanied by swollen regional lymph nodes, general discomfort, and fever [93].

A specific BPXV vaccine is not available. However, a live vaccine with an attenuated VACV strain can be used for prophylactic control and protection of animals in an infected flock. The appearance and reappearance of these OPXVs are alarming if one considers that around 50% of the world population over 30 years of age is not vaccinated against smallpox and is more vulnerable in the event of a reappearance of this disease.

## 11. Lumpy Skin Disease

The Lumpy skin disease virus (LSDV) is a double-stranded DNA virus belonging to the genus Capripoxvirus. It is closely related to goat and sheep pox viruses in the Chordopoxvirinae subfamily of the Poxviridae family [97,98]. The disease is known by various names such as “LSD”, “Pseudourticaria”, “Neethling virus disease”, “exanthema nodularis bovis”, and “knopvelsiekte”. LSD is a non-zoonotic, vector-borne disease, with a limited host range and currently restricted to ruminants viz. cattle and water buffaloes, which are particularly susceptible. The arthropod vectors responsible for the disease spread include biting flies, mosquitoes, and ticks [99]. Wild ruminants can act as potential reservoirs for disseminating the LSD virus. The emergence might be associated with climate change in different regions because LSD is an arthropod-borne transmissible disease.

Lumpy skin disease (LSD) is a viral transboundary or transcontinental disease endemic throughout Africa and in some parts of the Middle East and Europe and has increasing potential for global spread, with high economic impact that affects cattle and domestic water buffaloes [97,100]. The first outbreak of LSD was found in 1929 in Zambia, then spreading throughout Africa and with an ongoing expanding distribution to Asia and Europe. In 2019, the disease was reported for the first time in many bordering countries in South Asia, such as India, Nepal, China, and Bangladesh. In 2020, LSD was also recorded in Bhutan, Sri Lanka, Bangladesh, Vietnam, and southeast China [99]. LSD was also found in Southeast Asia, in Vietnam and Myanmar. In 2021, it further spread to new countries such as Thailand, Malaysia, Cambodia, and Laos, [98,101]. According to the OIE and WOAH report, at present, the disease is endemic in most African, European, and Asian countries [99]. After its first appearance in Zambia more than 90 years ago, the virus dispersed to numerous locations worldwide.

In recent years, LSD has become one of the most devastating and appearing threats to large ruminants such as domestic cattle, water buffalo, and wild bovine species. The World Organization for Animal Health (WOAH) includes LSD as one of the most economically meaningful and notifiable transboundary viral animal illnesses [102].

The disease can emerge several hundred kilometers away from initial (focal) outbreak sites within a brief period. These attacks have initiated a long-awaited, revived scientific interest in LSD, resulting in novel research into broad elements of the disease, including epidemiology, modes of transmission, and associated risk factors. Long-distance dispersion of LSDV appears to occur through the motion of infected animals. However, distinct seasonal patterns indicate that arthropod-borne transmission is most likely responsible for the disease’s swift and aggressive short-distance spread [100].

Vector-borne disease transmission is most likely mechanical. To date, the most likely vectors for LSDV transmission are blood-sucking arthropods such as stable flies (*Stomoxys calcitrans*), mosquitoes (*Aedes aegypti*), and hard ticks (*Rhipicephalus* and *Amblyomma* species). New evidence suggests that the house fly, *Musca domestica*, may also play a role in LSDV transmission [100]. Wild ruminants, such as buffaloes, are thought to play a role in the epidemiology of these diseases [97]. Hematophagous arthropod-borne mechanical transmission is considered primary and the most common route; however, other transmission routes related to illegal animal trade have played a role in the emergence of LSD in countries otherwise/earlier free from it. The LSD virus can be transmitted in saliva and milk and from infected skin lesions [97].

LSD generates tremendous economic losses, such as leather damage, diminished milk production, abortion, and death in infected ruminants [98]. LSD is characterized by fever; mastitis; orchitis; swelling of the peripheral lymph nodes; multiple, firm, characteristic circumscribed nodular lesions or skin lumps over the whole body; and necrotic plaques in the mucous membranes [97], and is occasionally associated with systemic signs. Although LSD has a low mortality rate, the development of lesions can cause complications [101].

No effective treatment against LSD has been developed. Anti-inflammatory drugs and antibiotics are used as symptomatic treatment. To control the disease, effective control and preventive measures need to be implemented, as follows: (a) Movement of infected animals with LSD should be strictly prohibited to prevent the spread of transboundary disease. Within countries, sick animals should be quarantined for inspection to prevent the rapid spread of disease. (b) Vector movement due to prevailing winds may cause disease transmission. Vector control methods including vector traps and insecticides can also be used for preventing the disease. (c) Vaccination: A live attenuated vaccine is available for LSD based on different strains of the LSD virus. Companies have prepared vaccines, including one based on the Neethling strain like Lumpy Skin Disease Vaccine for Cattle [99]. A live, attenuated vaccine based on sheep pox virus also has been used to fight LSD outbreaks [97]. Also, monitoring the situations of LSD outbreaks continuously by disease surveillance is important [101]. Studies suggest that Egyptian water buffalo serves as an accidental non-adapted host for the disease, and this point requires deeper investigation. In addition, the current vaccine strategy should be re-evaluated for more coverage and effectiveness [103].

The appearance of infections like LSD amid and after COVID-19 has further harmed the economy. Therefore, it must be reviewed to protect and support the backbone of the developing countries’ economy in Southeast Asia.

## 12. Schmallenberg Virus (SBV)

In Germany and the Netherlands in 2011, a new cattle sickness was described and associated with a novel RNA virus named Schmallenberg virus (SBV), similar to the German locality where the outbreak appeared [104,105]. The discovered SBV contains sequence similitudes to other viruses in the *Peribunyaviridae* family, genus *Orthobunyavirus*, like Akabane, Aino, and also, the bluetongue virus, an orbivirus, shares several aspects in epidemiology with SBV, such as the affected mammalian host species and the insect vector [105]. 

*Culicoides* spp. midges play a role in their transmission and are related to climate change. The mechanism by which the vector-borne SBV survives over winter has not been clarified yet; however, the potential mechanism of transovarial transmission in the *Culicoides* vector was demonstrated in a field study. The initial source of SBV remains unclear; however, some reports relate cross-reactive antibodies to other Simu serogroup viruses from African cattle [106].

In 2012, a report from the European Food Safety Agency (EFSA) showed that the virus had been reported in 14 Central European countries and Eastern Europe. Nowadays, SBV is considered endemic in Europe, and occasional outbreaks and virus circulation have been documented in several countries, including Mediterranean countries (such as Italy, Spain, and Greece) and Northern Europe [107]. SBV re-emerged in cattle and sheep in Germany and The Netherlands in late 2014 and more recently in Belgium, France, and the UK. SBV clinical findings have only been seen in domestic ruminants. In contrast, only indirect serological SBV proof has been reported in wild ruminants (e.g., alpaca, buffalo, deer, chamois, mouflon, bison), zoo animals (e.g., kudu, zebra, oryx), and some other mammalians (e.g., horse, wild boar) [106]. 

Few studies of SBV seroprevalence have been reported in water buffaloes. A study in Turkey using samples collected from slaughterhouse ruminants between 2006 and 2013, including Anatolian water buffalo, showed a seroprevalence of only 1.5%. It was suggested that cattle are more susceptible to SBV infection than buffaloes [108].

A study in Italy in 2020 showed high SBV seroprevalences of 38.2% (cattle) and 43% (water buffalo) using a commercial SBV ELISA, comparable to that obtained in other countries under post-epidemic conditions. A virus neutralization assay showed high titers in many animals, which is assumed to indicate recent exposure. Some environmental factors were associated with higher seroprevalence, such as mean annual temperature, distance from the coast, and altitude. Mean annual temperature was found to be the main factor responsible for the virus distribution in southern Italy [105]. No correlation between individual factors (species, age, and origin) and SBV positivity was found, although water buffalo and younger and purchased animals revealed higher seroprevalence [105]. This study is the first to assess the seroprevalence of SBV in Mediterranean water buffalo and their role in the SBV epidemiology. Environmental variables appeared to be implicated in the transmission of infection; greater mean annual temperatures were significantly related to increased seroprevalence [105]. In contrast, in a preliminary study in China, 4 out of 21 water buffaloes tested positive for the presence of antibodies using an SBV ELISA that allowed for the detection of multiple Simbu serogroups [105].

SBV in adult ruminants usually results in non-specific clinical manifestations. In cattle, SBV infection usually manifests as a mild and brief illness, with anorexia, hyperthermia, diarrhea and reduced milk yield—also, congenital defects in newborn calves, lambs, and kids with arthrogryposis and hydranencephaly syndrome [105,106].

Because of the similarity of clinical characteristics of SBV with those of different ruminant virus diseases, virological and serological diagnosis is needed to confirm the infection. Detecting anti-SBV antibodies in serum is an indirect method for diagnosing SBV infections. The enzyme-linked immunosorbent assay and virus neutralization test [106], and recently, real-time RT-PCR [105], have been used for serological diagnosis.

Vaccination of replacement stocks and control of insect populations are the two most important methods for preventing SBV outbreaks. Inactivated SBV vaccines have been developed in Europe to protect sheep and cattle. Recombinant modified-live SBV vaccines have been produced, conferring high levels of protection. Although these vaccines are compatible with DIVA (differentiating infected from vaccinated animals), security considerations linked to virulence reversion may discourage their broader usage [106]. 

Epidemiological data support the possible resurgence of SBV infections when favorable conditions exist in some countries, including numerous naïve susceptible animals and augmented vector densities [106]. An unresolved issue is how the virus is preserved during winter when the vector population is scarce or nonexistent. Future studies are necessary to elucidate the probability of SBV introduction/re-introduction via infected midges imported from tropical or endemic regions with serological, molecular, and vector prevalence [106]. It is a fact that water buffaloes are suitable hosts for viruses transmitted by *Culicoides*, as they prefer wet, swampy environments near water sources (often used for environmental enrichment). Recent works have anticipated that SBV will re-emerge; hence, it represents a constant threat to ruminant populations.

## 13. Papillomatosis

Bovine papillomatosis (BP) has been reported in buffaloes in India [109] and Italy [110], caused by bovine papillomavirus (BVP). Ten different genotypes of bovine papillomavirus (BPV) have been identified, from BPV-1 to BPV-10. BPV-1 and BPV-2 are associated with fibropapillomas in cattle [110]. BP is distributed worldwide in cattle but is relatively less common in buffaloes and infects cutaneous and mucosal epithelia, causing hyperplastic lesions.

The first report describing the histopathology and ultrastructure of cutaneous, perivulvar, and vulvar fibropapillomas associated with BPV-1 infection in the water buffalo by TEM and PCR and the expression of E5 in the fibropapillomas was in Italy in 2009 and considered an example of cross-species infection from cattle to buffalo by BPV-1 [110]. However, once infection of BPV is established in buffaloes, it spreads from buffalo to buffalo, with no intermediate involvement of cattle [111].

The first confirmed report on the incidence of cutaneous warts (CWs) in water buffaloes associated with BPV-2 was in India in 2010 [109]. Bovine papillomavirus (BPV)-like particles were identified by negative staining and transmission electron microscopy (TEM). BPV-2 was detected by PCR and confirmed by nucleotide sequencing and phylogenetic analysis. Lesions were described as cauliflower-like or dome-shaped lesions, diagnosed as fibropapilloma/papilloma, and established as a new disease entity [109].

Fibropapillomas in buffaloes are described on the back, gluteal, vulvar, and perivulvar regions, common sites of scraping and scratching, and it is known that papillomavirus tumors tend to manifest at the site of trauma, probably caused by the release of inflammatory cytokines, reactivation of the latent virus, and cell proliferation [110].

The first report on ruminal papillomatosis in buffaloes in India and worldwide was associated with BPV-5, -1, -2 by quantitative real-time PCR [112]. Grossly wart-like lesions in the rumen, reticulum, mouth, and esophagus revealed small nodular to larger spherical or slender growths with a thin base on the mucosa and ruminal pillar. Histopathologically, they were diagnosed as fibropapilloma/papilloma.

More recently, Jangir et al., in 2017 [111], also in India, studied the pathomorphology and association of different BPV types in buffalo cutaneous warts, revealing that growths were either single or multiple, variable in shape and size, and at different body sites. Histopathologically, they were categorized as papilloma, fibropapilloma, and fibroma. PCR revealed the presence of BPV-1, -2, and -5 DNA. It was the first time BPV-5 DNA was detected in buffalo CWs in India. In conclusion, DNA of BPV-1, -2, and -5 was detected either alone or in mixed infections.

Interestingly, other authors describe thirteen bovine papillomavirus (BPV) types (BPV-1 to -13) [113]. Apart from BVP 1–2 and 5 related to cutaneous warts or gastrointestinal lesions, BPV-2 is known to play a central role in bladder carcinogenesis of adult cattle reared on pasturelands rich in bracken fern (*Pteridium aquilinum*). Tumors of the urinary bladder of buffaloes have been only sporadically reported. Roperto et al. was the first to reveal an association between BPV-2 infection and tumors of the urinary bladder in buffaloes reared on lands rich in bracken fern in Turkey, demonstrating that BPV-2 is involved in buffalo bladder carcinogenesis, too. However, BPV-2 DNA was also found in non-neoplastic and healthy urinary bladder lesions. The results indicated that a latent infection of BPV-2 may occur in the bubaline normal urothelium [113]. Further virological and epidemiological studies are necessary to understand the incidence of BVP-2 infections in buffaloes and their emerging role in neoplastic and non-neoplastic diseases.

No effective therapy is currently available for BPV. Traditional attenuated or killed vaccines from cultured BPVs are not sufficiently effective. Different therapies have been implemented in field conditions in bovines, such as surgical removal of warts, cauterization, immunotherapy, and homologous vaccines. However, effective prophylactic vaccination against BPV is still not available. Virus-like particles represent a promising vaccine platform for a diverse array of viruses. These particles mimic the morphological and immunological features of native virions, but cannot replicate and cause disease due to the absence of a viral genome [114]. No vaccination studies in buffaloes were identified.

## 14. Final Considerations

Due to their distinct anatomical and physiological characteristics, water buffaloes are often postulated to exhibit greater resistance to certain diseases than cattle. However, this review highlights that buffaloes can be just as vulnerable to specific viral diseases as cattle, with clinical presentations ranging from mild or subclinical to more severe forms.

A pivotal factor in the epidemiology of these viral diseases is the typical habitat of buffaloes: tropical, flood-prone zones. With their inherent hot and humid conditions, such environments, are ideal for the proliferation of disease-causing pathogens or vectors. In addition, the introduction and movement of buffaloes, even their biological products, among different continents, countries or regions have been implicated in disseminating exotic diseases in specific locales, notably Foot-and-Mouth Disease and Rinderpest. Diagnostic procedures in water buffaloes must duly incorporate these diseases, particularly given their obligatory reportable designations. In this regard, the notable expansion of buffalo populations in Latin America underscores the inherent risks linked to the introduction of external herds or biological products.

The ramifications of the climate change crisis for the epidemiology of viral diseases affecting buffaloes is another factor that cannot be understated. For instance, shifts in vector populations responsible for transmitting diseases, exemplified by Orbivirus-induced Blue Tongue, demand attention. The unpredictable nature of these diseases presents further complexities. There are instances where animals manifest symptoms suddenly after years of exposure, which is the case with Malignant Catarrhal Fever. On the other hand, for some diseases such as Bluetongue, BHV-1, and Bovine Viral Diarrhea, there are reports of a high seroprevalence in buffaloes in some regions, but this is accompanied by low disease incidence and mortality, thus exhibiting enzootic stability. Examining infection dynamics within these scenarios remains a paramount priority for effectively preventing and controlling viral diseases in buffalo production systems.

In recent decades, the increasing emergence or recurrence of major transboundary and emerging animal diseases has evolved into a tremendous economic and public health concern worldwide, impacting food security by reducing the availability and affordability of high-quality animal products.

A key issue is that diagnostic tests should be implemented since there are no tests dedicated to this species; furthermore, it is crucial always to verify the applicability of the tests developed on cattle, including a study dedicated to the buffalo species.

Lastly, the discovery of new viral variants with the potential for interspecies transmission—whether between cattle, buffaloes, or humans—highlights the importance of understanding host–pathogen dynamics. The current SARS-CoV2 pandemic, juxtaposed with diseases like buffalopox, underscores the broader susceptibility to emergent viral pathogens. Continuous surveillance of viral strains in buffaloes remains critical, given the potential risks of new variants crossing species barriers.

## 15. Conclusions

The evolving epidemiology of buffalo viral diseases highlights the imperative for ongoing surveillance and adaptive research in developing and implementing effective prevention and control programs. Given the complex interactions among buffaloes, co-existing species, pathogens, and the shared ecological environment, a holistic and interdisciplinary approach is essential. Such a comprehensive strategy promises the creation of robust health protocols and proactive disease management measures, adaptable to the ever-changing environmental and biological challenges.

## Data Availability

The data presented in this study are available in article.
